# Personality network neuroscience: Promises and challenges on the way toward a unifying framework of individual variability

**DOI:** 10.1162/netn_a_00198

**Published:** 2021-08-30

**Authors:** Kirsten Hilger, Sebastian Markett

**Affiliations:** Department of Psychology I, Julius-Maximilians University Würzburg, Würzburg, Germany; Department of Psychology, Goethe University Frankfurt, Frankfurt am Main, Germany; Department of Psychology, Humboldt-Universität zu Berlin, Germany

**Keywords:** Personality network neuroscience (PNN), fMRI, Personality psychology, Trait theory, Functional brain connectivity

## Abstract

We propose that the application of network theory to established psychological personality conceptions has great potential to advance a biologically plausible model of human personality. Stable behavioral tendencies are conceived as personality “traits.” Such traits demonstrate considerable variability between individuals, and extreme expressions represent risk factors for psychological disorders. Although the psychometric assessment of personality has more than hundred years tradition, it is not yet clear whether traits indeed represent “biophysical entities” with specific and dissociable neural substrates. For instance, it is an open question whether there exists a correspondence between the multilayer structure of psychometrically derived personality factors and the organizational properties of traitlike brain systems. After a short introduction into fundamental personality conceptions, this article will point out how network neuroscience can enhance our understanding about human personality. We will examine the importance of intrinsic (task-independent) brain connectivity networks and show means to link brain features to stable behavioral tendencies. Questions and challenges arising from each discipline itself and their combination are discussed and potential solutions are developed. We close by outlining future trends and by discussing how further developments of network neuroscience can be applied to personality research.

## INTRODUCTION

A major goal of psychology is to understand how and why people differ in thought and behavior—and how such differences are organized in an individual’s personality. The foundation of personality psychology is the observation that individual differences follow principles, that is, traits or dispositions, that are sufficiently stable within individuals, sufficiently consistent between individuals, and sufficiently invariant to situational context to explain past and to predict future behavior. Traits are hierarchically organized constructs that in their entirety constitute a multidimensional space where each individual can be placed to describe their personality and thus individuality (Allport, 1937; Eysenck, 1947; Guilford, 1959). The main assumptions and implications of the trait concept as well as its presumed neurobiological foundation will be introduced in the following section.

### Main Conceptions of Human Personality

**Figure 1.**
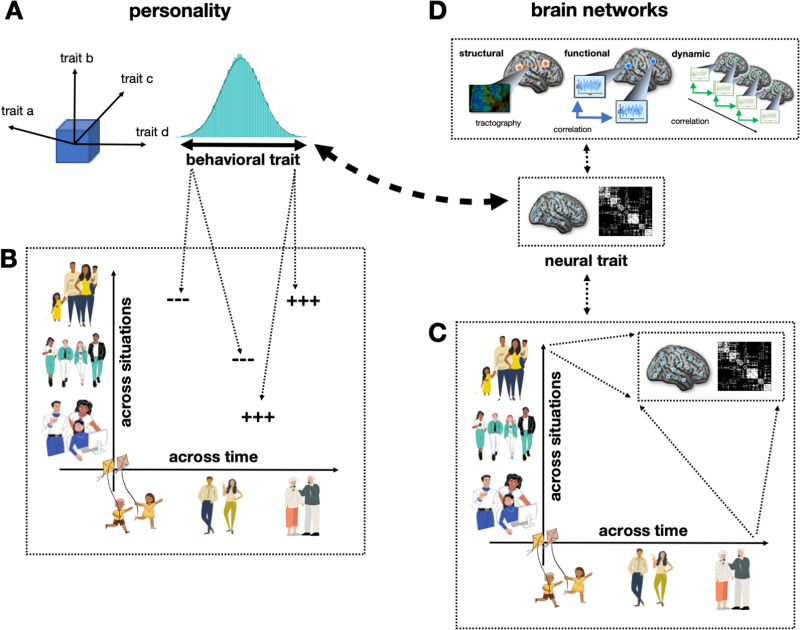
Schematic illustration of linking network neuroscience to personality research. (A) Personality is a multidimensional factorial space where traits are organized as bipolar dimensional constructs (e.g., introversion vs. extraversion). Each individual can be classified along the trait continuum, and the relative position compared to others is highly stable across time and situations. (B) Traits produce consistent behavior across (trait-relevant) situations and time. A person scoring at the trait’s upper end will respond consistently stronger (+++) to relevant stimuli than a person at the lower end (---). (C) Each situation is associated with a certain brain state (functional connectivity, network state). Such states bear a traitlike connectivity component that is consistent across (trait-relevant) situations and time. (D) These traitlike connectivity components represent the neural trait system, and presumably consist of structural, functional, and dynamic network characteristics. Mapping these components to personality traits (big dashed line) is the key objective of personality network neuroscience (PNN).

The most renowned example for a taxonomy of personality traits is the five factor model (Goldberg, 1990) with the basic traits neuroticism, extraversion, openness to experience, agreeableness, and conscientiousness. The Big Five are based on the “lexical hypothesis” stating that fundamental personality traits have left their trace in language and can be uncovered through a purely data-driven approach capitalizing on similarities between different adjectives in the dictionary (Allport & Odbert, 1936; Cattell, 1945). In general, trait theory views traits as orthogonal and dimensional constructs that allow for each individual to be positioned in a multidimensional trait space (Figure 1A). Traits produce consistent behavior across (trait-relevant) situations. A person scoring at the upper end of a given trait (e.g., extraversion) will respond consistently stronger to relevant stimuli (e.g., social cues) than a person with lower scores on the respective trait (Figure 1B). Since its beginning, trait theory has posited that traits are more than statistical abstractions of behavior and have a foundation within the human brain, that is, neural systems whose interacting elements constitute the neural equivalent of traits—a conceptual nervous system (Allport, 1937; Gray, 1972; Hebb, 1955; Pickering, 1997), and different lines of research have approached personality traits from a neurobiological perspective (Corr, 2004; Gray, 1972; see Box 1).

**Box 1.**  *Milestones of Personality Psychology from a Neuroscience Perspective*

- **Gordon Allport (1937)**:
  ○  Introduction of the “trait” concept
  ○  Traits as “biophysical entities” (brain systems)
- **Hans Eysenck (1947)**:
  ○  Neuroticism (emotional stability) and extraversion (stimulation seeking) as the major personality traits (revealed by factor analysis)
  ○  Neurobiological correlates of traits
  ○  Eysenck Personality Questionnaire (EPQ)
    ■  Still frequently used (e.g., in the UK Biobank)
- **Joy P. Guilford (1959)**:
  ○  Hierarchical organization of personality traits
    ■  Primary traits determine subordinate traits that in turn determine behavior
  ○  Identification of five traits by factor analyses
  ○  Paving the way toward the Big Five personality taxonomy
- **Jeffrey Gray (1972)**:
  ○  Reconceptualization of Eysenck’s traits based on neuropharmacology and brain lesion studies in animal models
  ○  Essential role of approach and avoidance behavior
  ○  Reward Sensitivity Theory:
    ■  Two brain systems: The behavioral activation system (BAS) with striatal and thalamic substrates and the behavioral inhibition system (BIS) within septohippocampal involvement
- **Robert Cloninger (1986)**:
  ○  Hierarchy of traits:
    ■  Temperament traits: Innate dispositions for approach behavior, avoidance behavior, and social reward (novelty seeking, harm avoidance, reward dependence, persistence)
    ■  Character traits: Traits develop through interaction of temperament traits and the individual learning environment (self-directedness, cooperativeness, self-transcendence)
  ○  Temperament and Character Inventory
    ■  Widely applied in neuroimaging studies, including network neuroscience investigations
- **Paul Costa & Robert McCrae (1992)**:
  ○  The Big Five:
    ■  Neuroticism, extraversion, openness to experience, agreeableness, and conscientiousness
  ○  NEO personality inventory (NEO-PI) and the shorter NEO five-factor inventory (NEO-FFI)
    ■  Applied in numerous neuroimaging investigations (e.g., in the Human Connectome Project)
- **Jaak Panksepp (1998)**:
  ○  Affective Neuroscience Theory:
    ■  Separable subcortical circuitry for primary affect and motivation (revealed by invasive brain stimulation and lesioning)
    ■  Neural circuitry operates as trait system and forms the basis of human personality
  ○  Affective Neuroscience Personality Scales (ANPS):
    ■  Personality questionnaire based on neuroscientific data rather than statistical aggregation of lexical data
- **Jeffrey Gray & Neil McNaughton (2000)**:
  ○  Revised Reward Sensitivity Theory:
    ■  Anxiety and fear as separable neural systems, and hence different personality traits
  ○  r-RST questionnaire distinguishes individual differences in the BAS, the BIS, and the flight-fight-freezing system (FFFS)
- **Kibeom Lee & Michael Ashton (2004)**:
  ○  Extensive reanalysis of previous factor analytical data sets
  ○  Claim: Big Five system should be amended by a sixth factor “Honesty-Humility”
  ○  HEXACO questionnaire as alternative to the NEO-FFI (widely used in psychology)

More specifically, trait theory presumes that different traits dissociate into independent neural substrates that represent different traits at the brain level and whose between-person variations map directly onto variations in the respective behavioral trait. Furthermore, trait theory posits that these neural substrates are characterized by the same traitlike properties as behavioral traits such as invariance across situations and time (Figure 1C) and that they directly influence ongoing information processing and behavior. It is proposed that each situation is characterized by a unique brain state consisting of (a) a neural reaction elicited by the physical aspects of the stimulus and (b) an idiosyncratic component associated with the individual trait level (Figure 1D). Finally, trait theory assumes that the idiosyncratic component that reflects the trait on the neural level overlaps with the neural systems responsible for the processing of trait-congruent situations (e.g., social and reward-related brain circuitry in the case of the trait extraversion). This overlap becomes apparent in neural signatures of ongoing information processing and can, thus, be investigated via neuroscientific methods during trait-congruent situations. However, trait theory also assumes that traits are more than the sum of associated behavioral and neural states. Parts of the neural trait systems will therefore persist detached from ongoing information processing states and should be accessible also by different approaches than task-constrained neuroimaging (e.g., via resting-state fMRI).

In the following we argue that the mapping of structural, functional, and dynamic network characteristics of personality traits—a framework that we term personality network neuroscience (PNN)—holds exciting prospects to test key hypotheses from trait theory and to ultimately identify such neural trait systems. Before we summarize the current state of the art and outline how the connectome paradigm (Hagmann, 2005; Sporns et al., 2005) and network neuroscience with its rich methodology such as graph theory (Bassett & Sporns, 2017) can contribute to personality science more in detail, we will introduce the most common existing approaches toward measuring personality traits.

### Measuring Personality Differences

Personality traits are relatively broad dispositions that produce consistent behavior across a range of different situations. While it would in principle be possible to infer an individual’s personality from observed behavior, the current standard toward personality assessment is the use of psychometric questionnaires. Such questionnaires consist of a list of standardized test items, which are selected carefully to cover the trait’s most relevant aspects, to distinguish properly between individuals, and to achieve highly similar results across different assessments of the same person. Usually, each items consists of a short statement that is rated by the test takers as how well it describes themselves, for example, “I am someone who worries a lot” (indicative of neuroticism). Different assessment systems for personality traits have been proposed with varying length. Longer questionnaires allow for more accurate and reliable assessments, but can be a burden for the participant. In general, it is advised to select the minimum number of items so that the total questionnaire score allows for confidential generalization to a person’s personality trait and to future behavior.

It is, of course, imperative to psychometrically validate personality questionnaires, that is, to demonstrate the internal consistency of questionnaire items, to establish temporal stability (reliability), and to show theoretically meaningful associations with other data sources (external validity). This can, for instance, be achieved through peer ratings (assuming that close friends and relatives have similar abilities to rate a person’s personality) and behavioral experiments (Markett et al., 2014; Perkins et al., 2007, 2009). Relying on (standardized) self-report personality assessments is, however, not without criticism (Furnham, 1986). Alternative assessment approaches to personality that harness the rich behavioral and personal data from social media sites and smartphone-based assessments show promising results (Kosinski et al., 2013; Marengo & Montag, 2020; Stachl et al., 2020), but until these attempts overcome the proof-of-principle stage, self-report questionnaires remain the gold standard to assess a wide range of behavioral dispositions in the most effective way.

## PERSONALITY NETWORK NEUROSCIENCE

The goal of PNN is to identify and to integrate neural systems (or biophysical entities) associated with psychological trait conceptions within an integrated framework for human personality. We propose that such an integrative framework may pave the way toward a more mechanistic understanding of stable behavioral differences and we suggest that efforts toward this goal will include (a) the identification of stable and traitlike individual differences in brain network organization (*existence* of neural trait systems), (b) the mapping of these neural trait systems onto known personality traits (*associations* between neural traits and personality trait systems), (c) the demonstration of how these neural trait systems delineate in the sense of independent psychological traits and how the hierarchy and abstraction of personality traits is reflected in the structure of the neural trait system (*independence* of neural trait systems and *trait-congruent organization*), and (d) the investigation of how these neural trait systems lead to differences in behavior and trait-congruent responses to external stimulation (neural traits *predict* trait-congruent behavior). These objectives were mostly addressed only in isolation by previous research. Regarding case (a), for example, individual deviations from group-level networks in the form of reliable and stable network variants have been observed across different task states (Seitzman et al., 2019). Regarding case (b), several studies have demonstrated that personality traits can explain individual differences in task-evoked and intrinsic (resting-state) connectivity and activity (e.g., DeYoung, 2010; Dubois et al., 2018; Hsu et al., 2018; Markett et al., 2016; Mulders et al., 2018; Tompson et al., 2018; Toschi et al., 2018). Regarding case (c), studies mapped hierarchies and functional dissociations in affective and motivational brain systems to different personality factors (Mobbs et al., 2007), and similar personality profiles were linked to the similarity of brain connectivity patterns (Liu et al., 2019). Regarding case (d), a particularly promising approach—intersubject representational similarity analysis—attempts to dissociate stimulus-elicited neural activity with small between-subject variance from idiosyncratic and trait-related neural activity patterns (for review, see Finn et al., 2020). Specifically, this is done by residualizing neural time series from cross-subject correlations and linking the remaining similarities in neural time series to similarities in personality traits (Finn et al., 2020). This approach is based on intersubject correlation analysis (e.g., Gruskin et al., 2020) but can, in contrast to the latter, also operate on the level of a single subject. Intersubject representational similarity analysis has been successfully applied to fMRI during tasks (Rhoads et al., 2020; van Baar et al., 2019) and during naturalistic viewing (Chen et al., 2020; Nguyen et al., 2019) and complements other approaches that investigated relationships between trait levels and brain responses during trait-congruent situations (Perkins et al., 2019).

Importantly, the identification of idiosyncrasies in brain structure and function and the confirmation of statistical relationships between organizational principles of the brain and behavioral differences are not sufficient to describe a trait system in the form of a conceptual nervous system as proposed by trait theory, that is, composed of dissociable neural systems that are stable over time and influence behavior in trait-congruent situations. We state that this would require that different lines of evidence are integrated into a common framework. It would, for instance, be necessary to show strong correspondence between idiosyncratic network variants and psychometrically measured personality traits. Psychometric traits are commonly based on questionnaire items that provide relatively good insights into the traits’ nature. Since this is not yet the case for neuroimaging-derived networks variants, establishing such correlations is a valuable step to aid interpretability. Furthermore, it will be necessary to test whether actual behavior in trait-congruent situations can be predicted from idiosyncratic aspects of brain organization and to determine neuroanatomical borders for different trait systems. While multimodal association areas seem to carry the most idiosyncratic information about individuals (e.g., Finn et al., 2015), trait systems are also likely to include brain regions implicated in the processing of trait-relevant information, for example, regions associated with social and reward-related information in the case of the trait extraversion.

Of note, although neural networks can also be derived from electrophysiological (e.g., Langer et al., 2012), structural (e.g., Privado et al., 2017), and many other neural data sources, this perspective article will focus on functional networks modeled on the basis of functional magnetic resonance imaging (fMRI). Inherent limitations and methodological challenges that come up with introducing fMRI-based network neuroscience to personality research will be discussed briefly in the next section.

## CHALLENGES TO PERSONALITY NETWORK NEUROSCIENCE

### Challenges Arising from Personality Conceptions and Its Measurements

Questionnaire data can be collected alongside neurological data to map individual variation in personality traits to individual variation in, for example, brain network organization. While such approaches are widely used to study the “neural correlates” of personality, their strictly correlative nature remains also their greatest limitation, particularly since naturally occurring individual differences cannot be controlled experimentally. Situational context, in contrast, can be systematically manipulated and can therefore inform whether implicated brain systems produce trait-relevant behavior and react differently to external stimulation in a trait-congruent way (Markett et al., 2018). Furthermore, it is important to be aware of the fact that the size of any such correlative association is inherently limited by the plausibly not perfect reliability of both the personality assessment and the neuronal measure of interest (Sui et al., 2020; Vul et al., 2009). Finally, any personality questionnaire relies on specific theoretical assumptions about how personality is structured within the human mind. Thus, any personality assessment can only be valid as long as these theories’ assumptions are met and the theory may reflect “reality” (construct validity).

### Challenges Arising from Network Neuroscience

Just like personality measures, measures of brain network interactions are also characterized by imperfect reliability. This applies to both the functional connectivity itself as well as to the measures calculated on the basis of the respective brain connections. Although single brain connections seem to be characterized by poor to medium reliability, the global pattern on whole-brain connectivity seems to be quite reliable (Noble et al., 2019; Termenon et al., 2016). Furthermore, specific parameters were identified that critically impact the reliability of functional connectivity estimates (e.g., localization of brain connections, Mueller et al., 2015; scan duration, Birn et al., 2013; Demetriou et al., 2018; Hallquist & Hillary, 2018), which demonstrates the necessity of parameter-dependent reliability estimations and suggests opportunities to improve on reliability.

Another attempt to increase reliability in functional connectivity research is to subsume the most fundamental brain network characteristics within specific mathematical measures, such as graph metrics, that can provide insights into preferred routes of neural communication and into the functional specialization of brain networks (Rubinov & Sporns, 2010). However, the reliability of graph measures is also far away from perfect. While Telesford et al. (2013) reported higher reliability for clustering, path length, global and local efficiency than for degree, Braun et al. (2012) differentiated between first-order metrics (computed directly on functional connections) and second-order metrics (computed on first-order metrics), and suggested higher reliability for the latter. Beyond graph metrics, additional measures aiming to capture fundamental aspects of functional brain connectivity have recently been proposed and represent promising candidates for gaining deeper insights into the neural reflection of personality differences. For instance, Seitzman et al. (2019) suggest *network variants* as capturing important traitlike aspects of functional connectivity, while Salehi et al. (2020) propose specific relevance of temporally fluctuating individual network parcellations. The advent of time-resolved (dynamic) connectivity offers opportunities over and above the investigation of static (time-averaged) networks. For example, graph metrics can also be derived from *edge functional connectivity* (Faskowitz et al., 2019), *bipartitions* may complement our understanding about dynamic communication between large-scale brain networks (Sporns et al., 2020), and *high-amplitude events* representing states of high brain-wide cofluctuation can be evaluated for their potential to capture meaningful between-person differences in information processing dispositions (Esfahlani et al., 2020). Finally, the method of *connectome embedding* allows one to map individual differences through brain structure to brain function and to ultimately link them to behavioral variations (Levakov et al., 2021).

Another significant challenge to network analyses arises from variations in fMRI preprocessing pipelines (e.g., global signal regression, motion correction; Parkes et al., 2018) and uncertainty about the definition of network nodes and edges (van Wijk et al., 2010). Although most often nodes are defined on the basis of anatomical or functional brain atlases, concurring approaches are also available (e.g., Gordon et al., 2016), and there is still no consensus about the optimal way of parcellating the brain into meaningful partitions. However, different parcellations result in networks of different size and density, which can critically affect the reliability and robustness of network measures and thus impede comparability across different persons, groups, and studies (Zalesky et al., 2010). Attempts to deal with this challenge imply computing measures on different parcellation schemes and the development of more standardized parcellation approaches (e.g., Schaefer et al., 2018). Similarly, there is uncertainty about the definition of network edges, for example, weighted versus unweighted, density thresholds for binary networks, parameters for sliding-window approaches in dynamic network analyses—most of them having a critical impact on the comparability of computed measures (Ginetstet et al., 2011; van Wijk et al., 2010). However, these challenges can also, in principle, be ameliorated through demonstrating the robustness of results across different edge definitions or by temporally unresolving the correlation metric (Faskowitz et al., 2019). Finally, the selection of graph metrics also varies largely between different studies, and the alignment from graph metrics onto neurobiological processes is far away from being clear. Recommendations about a minimum set of metrics that should be reported in every study (Hallquist & Hillary, 2018) as well as further research on the biological underpinnings of graph metrics (e.g., via task-state connectivity; Cole et al., 2021; Greene et al., 2020) may help to address these limitations.

To conclude, most research suggests that functional connectivity may—despite its inherent limitations—represent a valuable tool to study neural correlates of stable individual characteristics such as human personality (Dubois et al., 2018; Gratton et al., 2018; Mueller et al., 2015; Noble et al., 2019; Sui et al., 2020). The existence of new candidate measures and the advancement of innovative ways of dynamic network analyses complement the overall optimistic picture and highlight the need for further developing the transfer of these methods to personality science.

### Challenges from Combining Personality Research and Network Neuroscience

Some of the limitations and challenges outlined above become especially critical when investigating neural correlates of individual differences (in personality) as they can, when ignored or not properly addressed, induce spurious associations between neural and psychological measures. Already during preprocessing, for instance, motion correction can induce bias in individual difference analyses. If, for example, the numbers of excluded fMRI frames (scrubbing) or the numbers of model regressors (spike regression) (Parkes et al., 2018)—both potentially affecting connectivity measures in an unwarranted way (Yan et al., 2013)—vary systematically with the (personality) variable of interest, this would induce artificially associations between neural and psychological measures. Therefore, motion correction strategies retaining the same amount of data points across participants (e.g., linear regression of 24 motion parameters; Satterthwaite et al., 2013) and reducing the loss of degrees of freedom (e.g., ICA-AROMA; Pruim et al., 2015) are recommended for a network neuroscience of personality.

Another critical issue is the definition of a common node parcellation scheme. Although most connectivity measures require the same number of nodes across subjects to allow for comparisons, it is obvious that individuals vary in both their anatomical subdivisions and in the way their brains are functionally organized into different subnetworks or modules (e.g., Hilger et al., 2017). Although some software packages provide options to partially account for individual differences in morphological characteristics (e.g., Freesurfer), the applied common parcellation scheme may still be more similar to the “true” individual partition in some subjects than in others. When the variance of the amount of this difference (individual partition vs. common partition) is not completely orthogonal to the variation in the individual difference measure, this can potentially confound the association between computed connectivity measures and the (personality) variable of interest. Recent advances toward individual node parcellations may help to develop new ways of comparing connectivity measures by simultaneously accounting for these differences (Salehi et al., 2020).

Finally, graph-theoretical measures can be crucially influenced by individual differences in mean connectivity strength (van Wijk et al., 2010). Although such individual differences can be meaningful, they complicate the interpretation of network measures. This seems to be especially problematic for efficiency measures (and other shortest paths metrics; Ginestet et al., 2011), while other measures seem to be more robust against such variations. In binary networks this issue can partially be solved by the choice of a proportional threshold retaining the same number of network edges for all subjects (but note the danger of including more spurious connections in networks with low mean connectivity strength; van den Heuvel et al., 2017), while weighted network measures are inherently affected by variations in network density and do, therefore, always represent a mixture of topological differences in network structure and differences in network density. Both can give rise to meaningful associations with personality variations, but for different reasons. Thus, especially for PNN, it is recommended to compute graph measures on unweighted (proportionally thresholded) and weighted networks and to explore in both cases the effect of controlling for individual differences in mean connectivity strength (for a comprehensive discussion and recommendations, see Hallquist & Hillary, 2018).

## FUTURE TRENDS AND NEW DIRECTIONS

### Major Questions of Personality Psychology Addressed with New Methods

As outlined in detail above, PNN aims to identify and to integrate trait-related neural systems within an integrated framework for human personality. New connectivity-based methods such as connectome fingerprinting (Finn et al., 2015; Kumar et al., 2018), network variants analysis (Seitzman et al., 2019), and intersubject representation similarity analysis (Finn et al., 2020) provide a toolbox to relate idiosyncrasies in brain function to psychometric personality traits. Future work may extend these approaches by including the concurrent measurement of multiple personality traits, by studying a wider range of trait-relevant situations in the form of experimental tasks or naturalistic viewing paradigms (e.g., of trait-relevant movie scenes), and by predicting behavioral variability in new trait-relevant situations from neural data. Another important aspect is the demonstration of the persistence of neural systems (or biophysical entities) over time, inside and outside of trait-relevant situations: This may require additional approaches to investigate the relationship between task activations and structural connectivity (Medaglia et al., 2017) as well as between functional connectivity assessed during task and rest (Cole et al., 2021). Once neural systems (or biophysical entities) for different traits have been delineated, it will also become relevant to study their interaction. Although trait theory suggests different traits as dissociable independent systems, multiple traits are supposed to influence behavior in a given situation through complex interactions. Understanding these interactions will therefore be relevant to predict individual behavior in new situations.

### Open Questions and Further Developments

The network science of personality is currently restrained by pressing paradigmatic and methodological issues. Many imaging studies are still low in statistical power, particularly when it comes to brain-behavior correlations (Cremers et al., 2017). Possible solutions are the collaborative sharing of data and meta-analyses of published findings. Since the inception of the 1000 Functional Connectomes Project (Biswal et al., 2010), data were released from several thousand participants. Through other large-scale endeavors such as the Human Connectome Project (HCP), the Chimgen Project, and the UK Biobank, the number of included participants lies currently in the upper five digits. Such sample sizes are actually needed, as correlations between neuroimaging measures and psychological variables stabilize only in sufficiently large samples (Marek et al., 2020; Sui et al., 2020). The problem for personality research is that most available datasets do not contain a broad phenotypically (personality) assessment (except for the HCP that includes the NEO-FFI for its 1,200 participants), or if they do, make use of different tools (but see, e.g., Nooner et al., 2012). As long as they measure similar constructs, different assessment tools can be harmonized and combined—a strategy applied in molecular genetics where even larger sample sizes are required (Baselmans et al., 2019; Montag et al., 2020). Such strategies may also be viable for neuroimaging, particularly since it has been shown that brain-personality relationships generalize across datasets and psychometric approaches (Jiang et al., 2018). Also, further developments from the predominantly applied *explanatory* correlative approaches toward more *predictive* machine learning-based analyses (implying cross-validation) can further contribute to increase the reliability and reproducibility of findings when adopted on large samples (for an exemplary study in the PNN domain, see, e.g., Dubois et al., 2018). This, however, does only apply to situations where any personality data has been collected at all. Therefore, we would like to encourage researchers to include at least a basic personality assessment (e.g., the Big Five Inventory; John & Srivastava, 1999) into their projects, particularly when they plan to make their data publicly available.

Meta-analyses integrating results across published studies are a second route our field might consider. Cognitive neuroscience, for instance, has benefited tremendously from its meta-analytical approaches (Laird et al., 2005; Yarkoni et al., 2011). Classic cognitive neuroscience studies rely on a unified statistical framework (mass-univariate application of general linear models) within a universal topological space (Montreal Neurological Institute). Both are ideal preconditions for coordinate-based meta-analyses. Network neuroscience studies, on the other hand, show a wide variety in their basic approaches such as different brain parcellations, connectivity metrics, and resolution levels. A common reference frame for PNN investigations would be highly desirable.

Finally, the establishment of a link between the hierarchical modular structure of personality factors and the multilayer architecture of the human brain does, in principle, require a joint network approach, that is, the application of network measures on both neuroimaging and personality assessment data. Recently, pioneering studies from clinical psychology also demonstrated the usefulness of network analyses to questionnaire data (Duek et al. 2020; Taylor et al., 2020; for review, see Burger et al., 2020; Hofmann et al., 2020) and do thus suggest promising means—also for the further development of PNN.

### Toward an Individualized Precision Neuroscience

Finally, progress toward identification of neural trait systems (i.e., biophysical entities) and the mapping of these to psychological personality conceptions as outlined in this article would not only benefit psychology but would also be of key interest to psychiatry and clinical neuroscience. Personality traits overlap etiologically with psychiatric categories (Okbay et al., 2016), and manifestations at the extremes of individual variation play a key role in several conditions (Krueger & Markon, 2006). Furthermore, personality characteristics within the normal range are indicative for the choice of treatment and provide information about potential risk factors, suggesting their potential for the development of personalized medicine (Ziegelstein, 2017). Including personality assessments into new data collection and advancing a PNN could therefore also benefit the identification of clinically relevant biomarkers.

## AUTHOR CONTRIBUTIONS

Kirsten Hilger: Conceptualization; Project administration; Writing – original draft; Writing – review & editing. Sebastian Markett: Conceptualization; Project administration; Writing – original draft; Writing – review & editing.

## FUNDING INFORMATION

Kirsten Hilger, Deutsche Forschungsgemeinschaft (https://dx.doi.org/10.13039/501100001659), Award ID: HI 2185/1.

## TECHNICAL TERMS

Personality: Stable emotional, motivational, and cognitive dispositions that characterize a person’s individuality and explain his/her behavior across situations and time.
Conceptual nervous system: A part of the central nervous system that realizes a circumscribed psychological concept such as a personality trait.
Graph theory: Mathematical approach to model complex network systems as a set of nodes (e.g., brain regions) connected by edges (e.g., neural connections).
Psychometrics: A psychological discipline focused on theories and techniques to measure psychological constructs.
Internal consistency: A technical term from psychometrics that reflects how reliable an instrument measures a psychological construct.
Questionnaire items: An item is the fundamental unit of psychological assessment. A questionnaire item usually describes trait-congruent behavior and asks how this description applies to the test taker.
Intrinsic connectivity: Correlated neural activity measured during resting state, suggested to reflect intrinsically generated (as opposed to task-induced) functional brain networks.
Functional dissociation: A bidirectional one-to-one relationship between a specific neural substrate and a specific psychological function.
Naturalistic viewing: A more realistic, real-world-like stimulation in a cognitive neuroscience experiment (as opposed to a narrow focus on simple and controlled laboratory stimuli).
Graph-theoretical measures: Specific measures that allow one to characterize networks more in detail, thus providing insights into, for example, preferred routes of communication flow.
